# Adrenal Pheochromocytoma Treated With Stereotactic Body Radiation Therapy

**DOI:** 10.7759/cureus.12456

**Published:** 2021-01-03

**Authors:** Iván D González, Alexandra Vallejo, Eduardo Guerrero Lizcano, Alexandra Pabón Girón

**Affiliations:** 1 Radiation Oncology, Universidad Militar Nueva Granada, Bogota, COL; 2 Radiation Oncology, Instituto Nacional de Cancerología, Bogota, COL; 3 Medical Physics, Instituto Nacional de Cancerología, Bogot, COL

**Keywords:** pheochromocytoma, symptomatic control, metanephrines, sbrt

## Abstract

Pheochromocytoma is a rare neuroendocrine tumor arising from chromaffin cells in the adrenal medulla. In most cases, it is benign and tends to remain localized. However since it leads to the development of cardiovascular disease, it is associated with high rates of morbidity and mortality. Treatment options include medical, surgical, or ablative measures, which often adequately control the disease. Primary pheochromocytoma is conventionally treated with external beam radiation therapy (EBRT), while stereotactic body radiation therapy (SBRT) is preferred for cases with metastasis. However, literature regarding the use of SBRT for the treatment of primary disease is scarce. This case report describes a patient with an inoperable primary adrenal gland pheochromocytoma who was treated with SBRT, resulting in adequate symptomatic control during clinical follow-up.

## Introduction

Pheochromocytoma is a neuroendocrine tumor arising from chromaffin cells in the adrenal medulla. In most cases, it is benign and tends to remain localized [1]. It is considered a rare tumor with an incidence of two to eight cases per million inhabitants. Although it is a benign tumor, it is associated with high mortality and morbidities such as arterial hypertension and cardiovascular disease due to hypersecretion of catecholamines and metanephrines [2].

Germline mutations in susceptible genes account for approximately 40% of the cases. These include mutations in the VHL gene that codes for the von Hippel-Lindau protein. which possesses ubiquitin ligase 3E. It inactivates hypoxia-inducible factors that are involved in angiogenesis [3].

Von Hippel-Lindau disease is an autosomal dominant syndrome that affects 1 in 36,000 births per year. Unilateral or bilateral pheochromocytoma can occur in 10-20% of these patients; the mean age at diagnosis is 30 years, and approximately 5% develop metastatic disease at some point [4]. Overall, the 5-year survival rate in benign cases is 90 to 95%; however, malignant pheochromocytoma prognosis is poor, with a 5-year survival rate reported at 34 to 72% [5]. The classic triad of symptoms includes diaphoresis, headache, and tachycardia associated with hypertension and elevated catecholamines or metanephrines. Both plasma-free and 2-hour urinary fractionated metanephrines have more than a 90% sensitivity to pheochromocytoma diagnosis [6]. In addition, since 75% of these tumors are located in the adrenal gland, computed tomography (CT) or magnetic resonance imaging (MRI) of the abdomen or pelvis are appropriate imaging tools. On MRI, the classic imaging feature for pheochromocytomas is a “lightbulb bright” lesion with a solid hypervascular component in the T2 sequence. In T1 images, pheochromocytomas are typically isointense to muscle and hypointense to the liver. In the case of necrosis or hemorrhage, appearances can be quite variable [7].

There are several therapeutic strategies to treat this type of tumor. If the patient is asymptomatic, clinical follow-up can be done without therapeutic intervention. On the other hand, when the patient does have symptoms, multiple therapeutic approaches can be used for adrenal lesions, including systemic therapies, surgical treatment using open and laparoscopic adrenalectomy, Iodine-131, meta-iodobenzylguanidine (MIBG), or percutaneous radiofrequency ablation [8-9]. Additionally, antihypertensive medications are often required to prevent a catecholamine crisis and tumor lysis.

Using external radiation therapy to treat metastatic pheochromocytoma is controversial. Retrospective cohorts have shown that stereotactic radiosurgery/stereotactic body radiation therapy (SRS/SBRT) at a dose of 21.9 Gy provides symptomatic control or imaging stabilization at 81% and 87%, respectively [10]. Radiosurgery allows high doses of radiation with low toxicity to adjacent normal tissue to be administered [11]. The suggestion is to use SRS /SBRT in tumors less than 3 cm and normofractionated external beam radiation therapy (EBRT) for larger lesions [12]. The majority of reviews in available literature only partially document patient characteristics, radiation techniques, and results, making it difficult to establish a uniform treatment to follow and specific patient characteristics. There are multiple therapeutic approaches for adrenal gland lesions, such as stereotactic body radiotherapy/radiosurgery (SBRT), which provide an additional therapeutic option for patients with contraindications or who are refractory to the above-mentioned standard treatments. SBRT allows a high dose of radiation to be delivered with a rapid drop in the dose gradient to adjacent soft tissues.

Holy et al. analyzed effectiveness in patients in Germany and reported on 18 patients (13 with curative intent and five with palliative intent) treated with SBRT in doses that varied between 25 Gy in five fractions, 24 Gy in eight fractions, 36 Gy in six fractions, achieving a BED (Biological Equivalent Dose) of 37.5 Gy to 57.6 Gy. A local control rate of up to 77% was achieved [13].

The objective of this case report is to share the experience our institution had in adequately controlling the symptoms in a patient diagnosed with adrenal pheochromocytoma in whom other therapeutic alternatives were contraindicated. The patient was treated with SBRT, an innovative technique that delivers high doses of treatment to a small target while avoiding high doses to organs at risk.

## Case presentation

**Figure 1:**
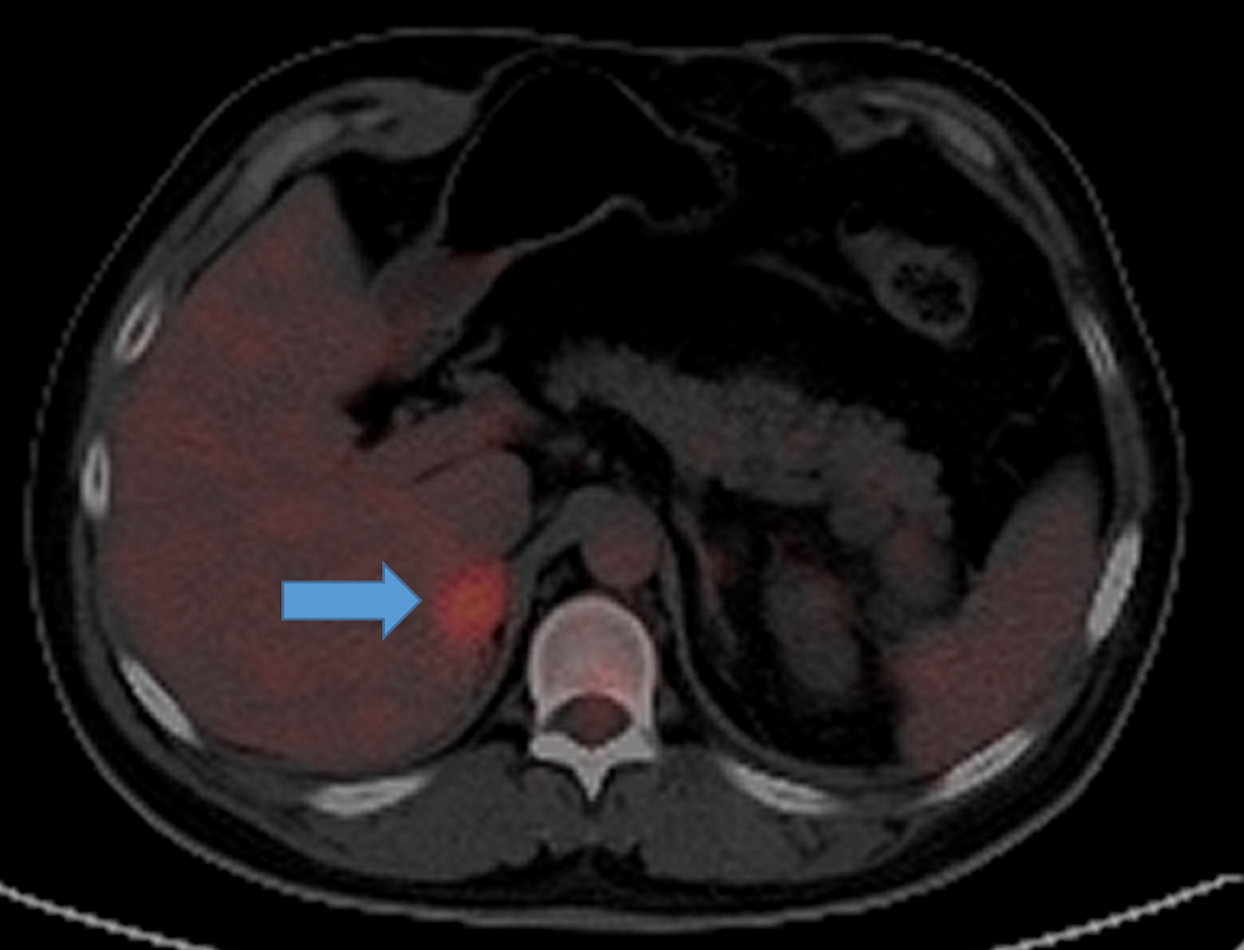
Positron emission tomography following 18F-FDG uptake An infiltrative, hyper-metabolic nodule dependent on the right adrenal gland can be seen in the image. It measured 24 x 19 mm and exhibited a soft tissue density and an SUV_max_ of 6. 8. SUV_max_: maximum standardized uptake value; 18F-FDG: 2-deoxy-2-fluoro(fluorine-18)-D-glucose.

A 27-year-old patient was diagnosed with von Hippel Lindau disease and had associated pathologies such as cerebellar hemangioblastomas treated with resection in June 2010, renal cell carcinoma diagnosed and treated with right nephrectomy in 2010, and a benign intra-adrenal pheochromocytoma (paraganglioma) on which a right-adrenalectomy was performed in 2011. Subsequently, the patient was treated with Sunitinib which was suspended due to cardiac toxicity in 2016. That same year a new right adrenal lesion appeared during clinical follow-up. In 2018 serum markers were found to be elevated, with free metanephrines in the plasma present at 110.8 pg/mL (normal value: 0 - 90 pg/mL) and free normetanephrines in the plasma present at 381.2 pg/mL (normal value: 0 - 180 pg/mL). mL), with a subsequent elevation of blood pressure requiring an increase in the dose of antihypertensive medications (enalapril, spironolactone, and doxazocin). In September 2018, a 24 x 19 mm hypermetabolic nodule located in the right adrenal gland was documented by positron emission tomography-computed tomography (PET-CT) (Figure 1). The case was discussed in a multidisciplinary meeting with the urology, oncology, interventional radiology, endocrinology, and radiation oncology departments where the conclusion was reached that the patient had no surgical choices due to comorbidities and technical difficulties presented in the previous surgeries, in addition to an associated coagulopathy. When evaluated by a radiation oncologist, the patient was on antihypertensive medication: 2 mg doxazocin at night, 5 mg enalapril daily (discontinuous intake), and 25 mg spironolactone daily. At that time, the patient was symptomatic with a headache rated 7/10 on the visual analog scale and poor control despite medical management.

**Figure 2:**
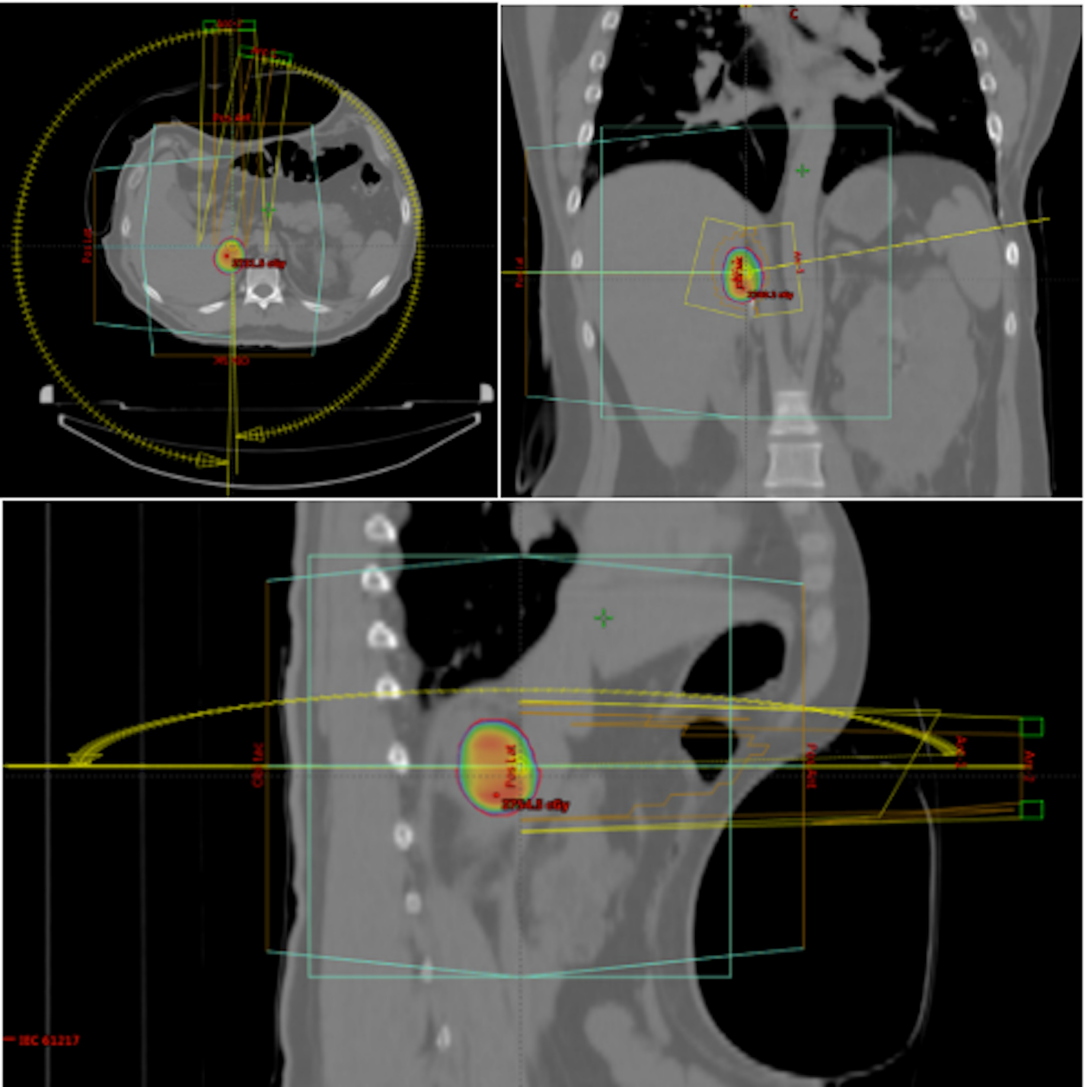
4DCT performed after stabilizing the patient with an abdominal compressor and a Vac-lock™ device The yellow and blue lines represent field disposition and arc conformation, respectively, in the axial, sagittal, and coronal planes (starting with the image on the top-left and moving counterclockwise). The color wash of the circular colored area represents the dose delivered to the target volume.

The medical board considered treatment using SBRT in fractionations of 800 cGy up to a total dose of 2400 cGy, which was to be delivered to the PTV (planning treatment volume = gross tumor plus a margin) to achieve local and symptomatic control. To delineate the tumor a 4DCT scan was performed after immobilizing the patient with an abdominal compressor and a Vac-lok™ device. Scanning was done in four respiratory phases followed by reconstruction of the images by 4D simulation of the 4DCT data. To delineate the tumor we fused the abdominal magnetic resonance and 4DCT simulation images of the patient lying in the treatment position (Figure 2).

Treatment volumes were then calculated as follows: CTV (clinical tumor volume) = GTV (microscopic tumor volume) + 2 mm volumetric expansion to encompass the macroscopic extent of the tumor PTV (planning treatment volume) = CTV + 5 mm margin

Radiotherapy was performed using two 6-MV non-coplanar VMAT (volumetric modulated arc radiotherapy) half arcs in the reverse direction. The arcs were modified using a multileaf collimator (MLC) to improve PTV (planning tumor volume) coverage and to reduce the dose to healthy tissue. An isotropic convergence beam with inhomogeneous dose distribution was used. Dose optimization was performed with the Photon Optimizer algorithm within the Eclipse™ system (version 13623) and dose calculation was performed using the Monte-Carlo method. The prescribed dose was normalized to 98% of the PTV isodose achieving a total of 1696 monitor units, a conformity index of 1.002, and a homogeneity index of 1.15. The maximum dose was 114% in the central part of the volume. The corresponding biological effective dose (BED) was 43.2 Gy.

The BED is calculated using the following formula: BED = dose per fraction x number of fractions x (1 + dose per fraction/[α/β]) In the formula above, the value of α/β is 10 Gy for tumor tissue.

A Clinac iX™ linear accelerator was used to deliver the radiation and patient-specific QA (quality assurance) was performed. The daily radiation fractions were administered under continuous blood pressure measurement to evaluate the endocrine function. Simultaneous image verification was performed with an onboard cone-beam CT imaging system. An abdominal compressor was used to improve the reproducibility of the position of the tumor target volume and to limit diaphragm movement. Treatment began on July 24, 2019, and ended on July 29, 2019, with in-hospital monitoring.

**Figure 3:**
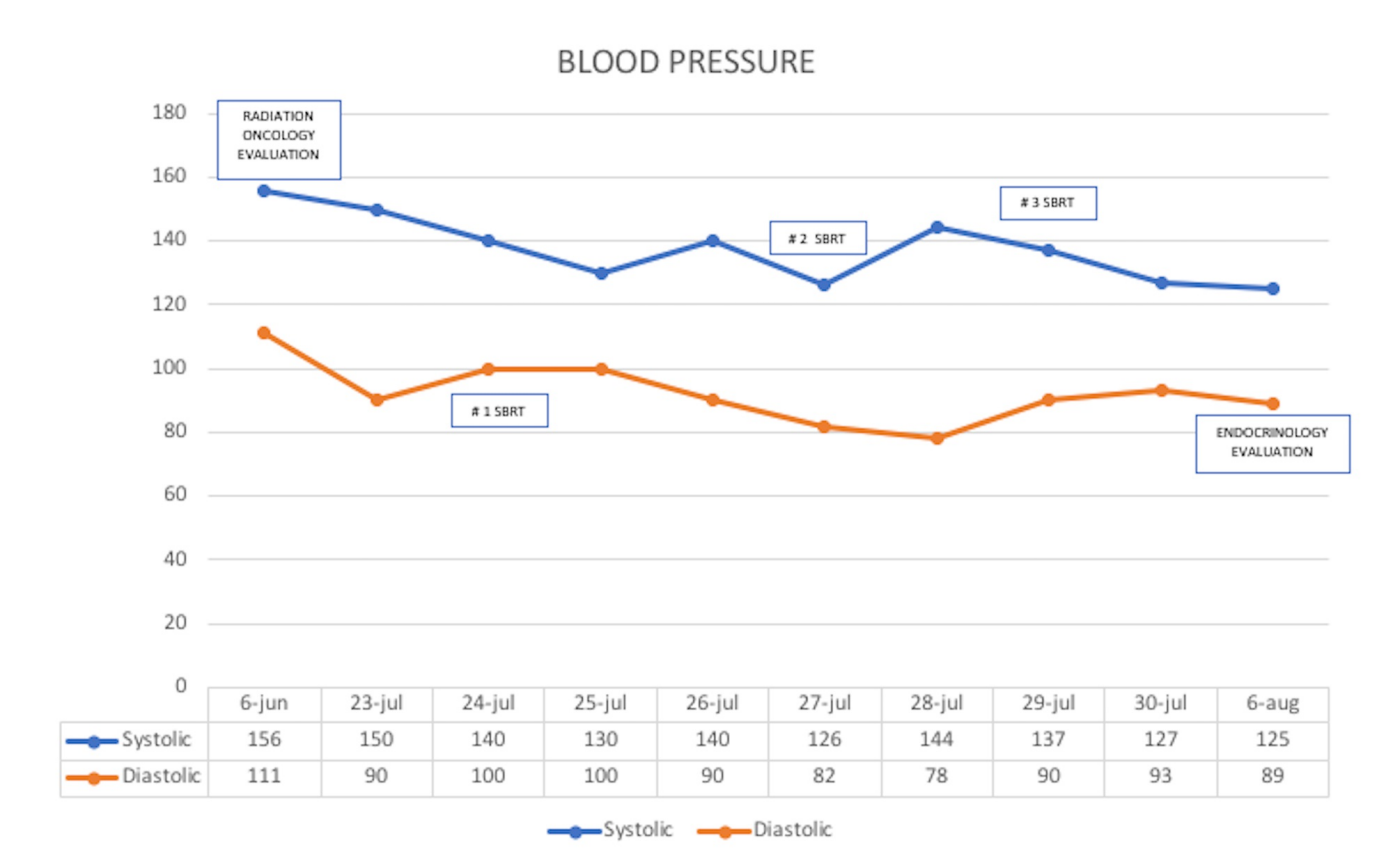
Blood pressure kinematics

The endocrinology group did the outpatient follow-up after the radiosurgery. Since the patient’s symptoms improved and blood pressure stabilized at 125/89 mm Hg (with mean arterial pressure, MAP, at 101 mm Hg), hypertension management with doxazocin, amlodipine, and hydrochlorothiazide was withdrawn and only carvedilol 12.5 mg was administered every 24 hours. There was no additional outpatient follow-up. However, the patient was admitted to the emergency service on August 15, 2019, with a medical history of a maximum-intensity right hemicrania headache radiating to the occipital region and associated nausea, despite being treated with seven morphine drops every eight hours, carvedilol 6.25 mg/day and levothyroxine 50 ug/inter daily. When admitted to the emergency room, the maximal blood pressure levels documented over the patient's medical history (Figure 3) at 148/106 mm Hg (MAP: 120 mm Hg) were observed, with a heart rate of 83 bpm, and pulse oximetry 87%. Later, the patient became hypotensive, had signs of dehydration, and was persistently hypoxemic. Therefore, intravenous fluids were ordered, a computed tomography scan of the head and magnetic resonance imaging of the brain were requested, and corticosteroid treatment was started. Despite the treatment, the patient started suffering generalized tonic-clonic seizures with supraversion and displayed central and peripheral cyanosis due to hypoxemia (he had 40% oxygen saturation according to pulse-oximetry). The diagnostic images taken did not show acute ischemic or hemorrhagic events but they did show nodular lesions suspected to be cerebellar hemangioblastomas, with no other significant changes compared to the previous studies. Despite management with phenytoin, the convulsive episodes and hemodynamic instability persisted with a tendency towards hypotension and hypoxemia. The patient was transferred to the resuscitation room. Clinical evaluation during the postictal state detected the absence of vital signs and death was declared on August 16, 2020.

## Discussion

Pheochromocytoma is a rare neuroendocrine tumor, which can be locally aggressive even when benign. It can be life-threatening due to elevated production of metanephrines by the adrenal glands and their direct impact on the cardiovascular system. The case described constitutes a therapeutic challenge by combining not only an aggressive presentation of the disease, but also a fragile patient with multiple comorbidities and contraindications for surgical and interventional treatments, limited pharmacological alternatives after presenting toxicity, and poor response to medical management.

Despite the scarce information available on radiotherapy treatment in cases of primary adrenal pheochromocytoma, there are numerous publications, mainly case studies (Table 1), which support the use of stereotactic body radiotherapy in adrenal metastases [9-13]. These studies report local control and adequate toxicities which justifies the use of this therapy in selected patients. Vogel et al., at the National Cancer Institute, published one of the largest reports on this topic. They found symptomatic control rates of up to 81% [10]; similarly, Breen et al. reported symptomatic improvement in 94% of patients treated with EBRT [1].

**Table 1:** Treatment doses used with conventional radiotherapy or SBRT to treat metastatic pheochromocytoma Fx: Fraction, BED: Biologically effective dose, PFS: Progression-free survival, OS: Overall survival, NSCLC: Non-small cell lung carcinoma, SRS: Stereotactic radiosurgery, SBRT: Stereotactic body radiotherapy, 3DCRT: 3D conformal radiation therapy, TPP: Time to progression, Mtx: Metastases, No. Fx: number of fractions.

Author	Country	n	Indication	Technique	Dose per Fx/Total Dose	No. Fx	BED	Local Control	PFS	OS (months)	Intention	Follow-up (months)
Holy et al. 2011 [13]	Germany	18	Adrenal Mtx & NSCLC Mtx	SBRT	8 Gy/40 Gy	5	43.2-72 Gy	77% (10/13)	2-42	21	13 curative	12-21
					5 Gy/25 Gy	5					5 palliative	
					8 Gy/24 Gy	3						
					6 Gy/36 Gy	6						
													Vogel et al. 2014 [10]	USA	3	Adrenal Mtx	3DCRT	1.8 Gy/54 Gy	30	No data	86.7%	22.47 (TTP)	52.4 (average)	Palliative	52.4 (median)
SBRT	8-8.5 Gy/24-25.5 Gy	3																							
Ippolito et al 2015 [14]	Italy	188	Adrenal Mtx & NSCLC Mtx	SRS	16-23 Gy	1	22.4-132 Gy	1 year: 44-100%	No data	12 (39.7 -90%)	Palliative	24
				SBRT	3-18 Gy/25-48 Gy	5		2 years: 27-100%		24 (13-53%)		

This case study adds to the available literature on the efficacy of radiotherapy for the treatment of adrenal pheochromocytomas to relieve symptoms and control blood pressure. Given the fragility and general condition of the patient and unstable blood pressure variables despite multimodal pharmacological management, and after taking into account the effectiveness of stereotactic body radiotherapy in controlling metastatic lesions and the absence of other treatment options, this was considered a highly conformational therapeutic modality that could protect adjacent healthy tissue. A 24 Gy dose was delivered in 8 Gy fractions. In addition, 4DCT simulation, and planning and verification of the dose delivered per fraction were also carried out by onboard Cone-Beam Computed Tomography (CBCT) imaging. Knowing the risk of hemodynamic decompensation and alteration in blood pressure associated with adrenal lesion treatment, continuous hemodynamic monitoring was done by endocrine specialists and anesthesiologists during the delivery of each treatment fraction and afterward.

The patient was hospitalized for follow-up and close surveillance during treatment, and remained asymptomatic during all phases of therapy, with no significant elevation of blood pressure. The patient’s hospital discharge was authorized on July 30, 2019, after the radiotherapy ended. During outpatient clinical control, blood pressure was normalized and headache relief was achieved, so the amounts and doses of antihypertensive drugs were reduced. Approximately 20 days after outpatient control the patient was admitted to the emergency room and died of unclear causes. However, during this hospital admission, no figures higher than 148/106 (MAP: 120 mm Hg) were documented, no brain MRI images reported an ischemic or hemorrhagic cerebrovascular event or significant changes compared to a study taken three months earlier. Although the patient displayed hypoxemia and seizures, no exact cause of death was recognized.

## Conclusions

Despite its limited occurrence in the literature, we propose implementing stereotactic body radiotherapy (SBRT) to treat primary adrenal pheochromocytoma in refractory patients, or in patients in whom other treatments are contraindicated, or in patients in whom associated life-threatening conditions are present as was the case in this study. More reviews are needed concerning the safety and effectiveness of SBRT when treating tumors of a similar nature and location.
